# Safety and Efficacy of Phacoemulsification With Trabecular Microbypass Stent W Implantation in Primary Angle-Closure Glaucoma

**DOI:** 10.7759/cureus.74878

**Published:** 2024-11-30

**Authors:** Kazuyuki Hirooka, Hiromitsu Onoe, Hideaki Okumichi, Yoshiaki Kiuchi

**Affiliations:** 1 Department of Ophthalmology, Hiroshima University, Hiroshima, JPN; 2 Department of Ophthalmology and Visual Science, Hiroshima University, Hiroshima, JPN

**Keywords:** efficacy, istent inject w, phacoemulsification cataract surgery, primary angle-closure glaucoma, safety

## Abstract

Background

Investigation of the safety and efficacy of phacoemulsification with trabecular microbypass Stent W implantation in patients with primary angle-closure glaucoma (PACG).

Methods

Between August and December of 2023, this prospective study evaluated PACG patients who underwent phacoemulsification with iStent inject W implantation. All patients were 18 years and older and were monitored for 6 months after surgery. Changes in intraocular pressure (IOP) and the number of glaucoma medications at 6 months after the surgery were defined as the primary endpoint, while the safety of the iStent inject W implantation in these patients was the secondary endpoint.

Results

Of the six patients in the study, one was male and five were female, with patient ages ranging from 62 to 83 years. Pre- and postoperative IOP (at 6 months) ranged from 12 to 18 mmHg and 9 to 17 mmHg, respectively, with only one patient exhibiting an increase in the IOP from 12 to 17 mmHg. The number of glaucoma medications decreased in all but one case at six months postoperatively. Neither hyphema nor an IOP spike was observed in the present study. There was no evidence of iStent inject W occlusion or malposition at six months postoperatively in any of the cases.

Conclusion

In patients with PACG, phacoemulsification with trabecular microbypass Stent W implantation was demonstrated to be a safe and effective procedure.

## Introduction

The primary cause of irreversible blindness globally is glaucoma, with the most common type being primary open-angle glaucoma (POAG). In contrast to POAG, primary-angle closure glaucoma (PACG) is associated with the closure of the anterior chamber angle by synechial or appositional approximation between the iris and the trabecular meshwork. This type of glaucoma is more aggressive and more likely to lead to blindness. It was reported that the global prevalence of PACG in 2013 was 0.5% [1]. The number of people worldwide with PACG was estimated to be 23.36 million in 2020, with the number projected to increase to 32.04 million by 2040 [1]. In 2010, bilateral blindness was estimated to affect 4.5 million people with POAG and 3.9 million people with PACG. By 2020, these numbers had risen to 5.9 and 5.3 million people, respectively [2].

It has been reported that the angle closure mechanism is associated with an increased lens thickness, with age additionally playing an important role [3]. Pupillary block is caused by a small eyeball with a thickened and forward-moving lens [3]. Therefore, to relieve the pupillary blockage, removal of the thickened lens (cataract surgery) is performed to deepen the anterior chamber. In PACG, although cataract surgery has an angle-widening effect, eyes with chronic iridocorneal apposition and elevated intraocular pressure (IOP) often have trabecular meshwork damage that makes it unlikely to be repaired by simply widening the angle [4]. In order to adequately and sustainably control the IOP in these eyes, glaucoma surgery may be necessary. Although substantial IOP reduction can be achieved by using traditional filtering surgeries, such as trabeculectomy and tube shunt, patients can also incur short- and long-term risks such as hypotony, diplopia, and infection [5]. A diverse group of relatively new procedures referred to as minimally invasive glaucoma surgery (MIGS) have been shown to play an important role in glaucoma treatment. Due to these procedures' ability to reduce both the IOP and IOP-lowering medications with limited or no disruption to the conjunctiva and sclera, they can help patients avoid the risks associated with filtration surgery [6,7].

The United States FDA has approved the iStent trabecular microbypass (Glaukos Co., San Clemente, CA, USA), and more recently, the second-generation iStent inject trabecular microbypass stent (Glaukos Co.). These procedures are indicated in patients with mild to moderate open-angle glaucoma, with these stents designed to create a direct route from the anterior chamber to Schlemm’s canal for the aqueous humor to bypass the damaged trabecular meshwork. For example, there is a wide flange at the base of the iStent inject W (Glaukos Co.) designed to optimize stent visualization and placement. However, in most countries, these devices are designated as “off-label” for PACG patients. In the present investigation, we examined patients with PACG and evaluated the safety and efficacy of phacoemulsification with trabecular microbypass stent implantation.

## Materials and methods

Patient selection

The study protocol for this prospective study, with no control group, was approved by the Institutional Review Board of Hiroshima University (Approval No. CRB2022-0012) and registered as jRCTs062230007 in the Japan Registry of Clinical Trials (jRCT). We aimed to recruit as many PACG patients as possible from April 2023 to December 2023. During this period, we analyzed six eyes from six PACG subjects who underwent phacoemulsification cataract extraction combined with iStent inject W implantation. This study was conducted in accordance with the principles of the Declaration of Helsinki. All patients consented to be included in the manuscript.

To be included in the analyses for this study, the eyes had to meet several criteria: patients must be ≥18 years of age; the posterior trabecular meshwork must not be visible for ≥180 degrees on gonioscopy, with or without peripheral anterior synechiae (PAS); there must be glaucomatous fundus abnormalities such as thinning of the rim in the optic disc or a defect in the retinal nerve fiber layer (RNFL) corresponding to a visual field defect; a cataract and a need for IOP reduction must be present; and there must be follow-up data available for 6 months. We excluded patients with severe glaucoma (mean deviation worse than -12 dB), PAS in the nasal quadrant precluding the placement of iStent inject W, corneal endothelium cell density below 1500 cells/mm^2^, or an IOP ≥25 mmHg.

Surgical technique

A temporal corneal incision was made prior to phacoemulsification. The intraocular lens (IOL) was implanted in the capsular bag following cataract removal. To enhance visibility of Schlemm’s canal, sodium hyaluronate was added to the anterior chamber after IOL implantation. Subsequently, the goniolens was placed on the cornea after tilting the patient’s head and the microscope to observe the nasal angle. Two iStent inject W devices, spaced one to two clock hours apart, were inserted into the nasal quadrant trabecular meshwork after identifying Schlemm’s canal. Goniosynechialysis was not performed even if PAS was present. Postoperatively, all patients were started on eye drops three to four times a day, including 1.5% levofloxacin (Nipro Co., Osaka, Japan) and 0.1% betamethasone (Shionogi & Co., Ltd., Osaka, Japan), and 0.1% bromfenac (Senju Pharmaceutical Co., Ltd., Osaka, Japan) twice a day for 3-4 weeks. If patients had been using glaucoma eye drops prior to surgery, these were discontinued. Subsequently, based on the postoperative IOP, glaucoma eye drops were gradually reintroduced on an as-needed basis.

Other study measures

Prior to the surgery, all patients underwent a baseline examination. The preoperative data collected included age at the time of surgery, gender, best-corrected visual acuity (BCVA), and mean IOP. At every visit, the IOP was measured using a Goldmann applanation tonometer. Gonioscopy was performed under dark conditions using a narrow light beam and a 4-mirror goniolens (Volk Optical Inc., Mentor, OH, USA). Automated perimetry was conducted using the Humphrey Visual Field 24-2 Swedish Interactive Threshold Algorithm (SITA) standard program (Humphrey Field Analyzer II; Carl Zeiss Meditec AG, Jena, Germany). Optical coherence tomography (OCT) of the RNFL was performed with an RTVue-XR Avanti (Optovue Inc., Fremont, CA, USA). Corneal endothelial cell density was measured using a specular microscope and autofocus device (Topcon SP-3000; Topcon Co., Tokyo, Japan).

Patients were required to return to the clinical center at least five times: at 1 day, 1 week, and 1, 3, and 6 months postoperatively. The data collected at all postoperative consecutive visits included IOP measurements, the number of glaucoma medications, BCVA, and complications. A 4-mirror gonio lens was used to confirm the position of the iStent inject W at one day and six months postoperatively. At baseline and six months after the surgery, Humphrey perimetry and corneal endothelial cell density were reassessed.

Primary and secondary outcome measures

Differences in the IOP and the number of glaucoma medications at six months were defined as the primary outcome measures, while the incidences of intraoperative and postoperative complications were defined as the secondary outcome measures.

## Results

Table 1 presents the clinical characteristics of the 6 eyes from the six patients enrolled in this study. The patients included one male and five females, ranging in age from 62 to 84 years, with baseline IOPs ranging from 12 to 18 mmHg.

**Table 1:** Demographic findings of each case. y: Year; R: Right; L: Left; IOP: Intraocular pressure.

Patient	Age (y)	Gender	Side (R/L)	Baseline IOP (mmHg)	No. glaucoma medication	Corneal endothelial cells (/mm2)
1	83	Female	L	15	3	2691
2	64	Female	L	15	2	2954
3	62	Female	R	16	0	2876
4	65	Female	R	16	4	2673
5	74	Female	R	12	2	1985
6	64	Male	L	18	0	2365

Two trabecular stents were successfully implanted in all eyes. None of the patients experienced any intraoperative complications such as iris prolapse, iris trauma, or iridodialysis. Intraoperative hyphema occurred in all cases immediately after the insertion of the iStent inject W into Schlemm’s canal. The progression of IOP and the number of glaucoma medications are presented in Table 2. Preoperative and 6-month postoperative IOPs were 15.3 ± 2.0 mmHg and 14.2 ± 2.8 mmHg, respectively (P = 0.51; paired t-test). At 6 months postoperatively, except for one case (patient 5), the IOP was the same or lower than that observed preoperatively. At 6 months, patient 5 resumed glaucoma medication. In all but one case (patient 6), the number of glaucoma medications decreased at 6 months postoperatively. No significant difference was observed in the mean number of glaucoma medications compared to the baseline (P = 0.18; paired t-test).

**Table 2:** Individual data for IOP change. Preoperative and six-month postoperative IOPs were 15.3 ± 2.0 mmHg and 14.2 ± 2.8 mmHg, respectively (P = 0.51; paired t-test). IOP: Intraocular pressure; d: Day; w: Week; m: Month.

Patient	IOP (no. of glaucoma medications)					
	Baseline	1d	1w	1m	3m	6m
1	15 (3)	15 (0)	15 (0)	13 (0)	14 (0)	14 (0)
2	15 (2)	11 (0)	12 (0)	12 (0)	14 (0)	15 (0)
3	16 (0)	12 (0)	12 (0)	12 (0)	10 (0)	9 (0)
4	16 (4)	15 (0)	14 (0)	15 (0)	16 (0)	16 (2)
5	12 (2)	15 (0)	13 (0)	14 (0)	14 (0)	17 (0)
6	18 (0)	24 (0)	18 (2)	18 (0)	14 (2)	14 (2)

Changes in corneal endothelial cell density are shown in Table 3. Preoperative and 6-month postoperative corneal endothelial cell counts were 2591 ± 360 /mm² and 2481 ± 326 /mm², respectively (P = 0.31; paired t-test). No significant decreases in corneal endothelial cell density or instances of hyphema or transient elevation of IOP were observed in any of the patients. No evidence of iStent inject W occlusion or malposition was observed at 6 months postoperatively in any of the cases. 

**Table 3:** Changes in corneal endothelial cells. Preoperative and six-month postoperative corneal endothelial cell densities were 2591 ± 360 cells/mm² and 2481 ± 326 cells/mm², respectively (P = 0.31; paired t-test). m: month.

Patient	Baseline (cells/mm^2^)	6m (cells/mm^2^)
1	2691	2205
2	2954	2810
3	2876	2673
4	2673	2832
5	1985	2124
6	2365	2239

## Discussion

This study evaluated patients with PACG and investigated the safety and efficacy of phacoemulsification when using iStent inject W implantation. The efficacy of phacoemulsification with iStent inject W in patients with open-angle glaucoma has been evaluated and reported in several other previous studies [8-10].

A significant decrease in the IOP after six months was reported by Deneri S et al., with an IOP of 13.88 ± 2.57 mmHg (P < 0.01) compared to the baseline IOP of 16.08 ± 3.27 mmHg [8]. The preoperative IOP was 16.2 ± 4.4 mmHg, while it was 12.5 ± 2.6 mmHg at 6 months postoperatively (P < 0.001) [9]. As recently reported by Morita S et al., there was a significant decrease from the preoperative mean IOP of 15.0 ± 2.8 mmHg to the 12-month postoperative value of 13.8 ± 3.3 mmHg (P < 0.01) [10]. Although two previous studies reported a significant decrease in the mean number of IOP-lowering medications from the preoperative values of 2.1 ± 1.4 [9] and 2.9 ± 1.4 [10] to the postoperative values of 0.5 ± 0.8 (P < 0.001) at 6 months and 1.7 ± 1.6 (P < 0.01) at 12 months, respectively, a third study reported no significant decrease in the mean number of IOP-lowering medications preoperatively (2.69 ± 1.03) and at six months postoperatively (2.06 ± 1.34) [8].

Currently, there are no published studies on the safety and efficacy of the iStent inject W implantation in PACG patients. However, two studies have investigated patients with PACG and examined the efficacy and safety of phacoemulsification with iStent inject implantation [11,12]. One of the studies found a significant decrease in the preoperative medicated IOP from 18.6 ± 4.7 mmHg to 14.9 ± 2.8 mmHg at 6 months after phacoemulsification with iStent inject implantation [11]. Furthermore, at 12 months postoperatively, the mean number of glaucoma medications was 0.25 ± 0.68 [11]. After 12 months of follow-up, a study by Salimi et al. reported a decrease in the IOP from 17.5 ± 2.7 mmHg to 13.0 ± 2.3 mmHg, and a decrease in the glaucoma medications from 2.2 ± 1.3 to 1.3 ± 0.8 [12]. Based on the results found in these two previous studies, the authors concluded that in PACG eyes, phacoemulsification with iStent inject implantation was more effective than phacoemulsification alone. In this study, we did not compare standard phacoemulsification with the efficacy of combined phacoemulsification with iStent inject W implantation in PACG patients. However, it is our assumption that phacoemulsification with iStent inject W implantation is indeed more effective than phacoemulsification alone.

In this study, hyphema was resolved by the first postoperative visit, although in all eyes there was blood reflux into the anterior chamber when the iStent inject W was well inserted in Schlemm’s canal. In our recent study on POAG eyes, hyphema was observed in 1 eye (1.8%) [9]. However, on postoperative day 1 in this study, there was no hyphema. In both POAG and PACG eyes, hyphema is a rare complication. A study of PACG eyes by Chen DZ et al. reported that iStent occlusion with the iris occurred in five eyes (31.3%) [11]. However, we did not observe iStent inject W occlusion with the iris in this study. In open-angle glaucoma eyes, the incidence of iStent inject occlusion has been reported to be between 0% and 4% [13-16]. Therefore, even in PACG eyes, we speculate that iStent inject W occlusion with the iris is not higher.

The present study had a few limitations. First, our study did not contain a phacoemulsification-only control group. Therefore, distinguishing the effect of iStent inject W implantation versus the cataract extraction was not possible. Second, the present study only evaluated 6 eyes. Therefore, to obtain more rigorous and definitive evidence, we will need to conduct larger-scale, multi-center trials.

## Conclusions

In conclusion, the results of this study demonstrate both the efficacy and safety of combined phacoemulsification with iStent inject W implantation in eyes with PACG. We believe that this indication can be expanded, as cataract surgery can open the corneal angle even in cases with a narrow angle.
